# Reliability of Evidence to Guide Decision-Making in the Use of Acupuncture for Postpartum Depression

**DOI:** 10.3389/fpubh.2022.942595

**Published:** 2022-07-14

**Authors:** Xiuwu Hu, Qian Fan, Li Ma, Rui Jin, Rui Gong, Xiaoying Zhao, Fenfen Qiu, Liang Zhou

**Affiliations:** ^1^Nanchang Hongdu Hospital of Traditional Chinese Medicine, Nanchang, China; ^2^Department of Acupuncture, Changshu Hospital Affiliated to Nanjing University of Chinese Medicine, Changshu, China; ^3^Tianjin University of Traditional Chinese Medicine, Tianjin, China

**Keywords:** evidence, decision-making, acupuncture, postpartum depression, overview

## Abstract

**Background:**

There is conflicting evidence on the effectiveness of acupuncture in the treatment of postpartum depression (PPD). This study aimed to assess previous systematic reviews/meta-analyses (SRs/MAs) on the effectiveness of acupuncture to treat PPD.

**Method:**

SRs/MAs regarding the use of acupuncture for PPD were identified from the establishment of digital databases to November 2021. The Assessing the Methodological Quality of Systematic Reviews 2 (AMSTAR-2) was applied to evaluate the methodological quality of included SRs/MAs. The Grades of Recommendations, Assessment, Development and Evaluation (GRADE) was utilized to evaluate the evidence quality for outcomes of interest.

**Results:**

Six studies that conducted quantitative syntheses were included. According to AMSTAR-2, the methodological quality of these SRs/MAs was critically low owing to limitations of items 2, 4, and 7. According to GRADE, no study included high-quality evidence and most studies included low-quality evidence.

**Conclusions:**

Acupuncture m be beneficial for PPD, however, due to limitations of current evidence and inconsistent findings, further studies are needed to provide stronger evidence to draw definitive conclusions.

## Introduction

Postpartum depression (PPD) is a mood disorder associated with childbirth, since its onset begins between the first day and 4 months after delivery (1). Typically, PPD occurs within 6 weeks postpartum and patients tend to recover in 3–6 months, while severe cases can persist for up to 2 years. The prevalence of PPD in first-time mothers is as high as 16% (2), and the recurrence rate of PPD in the second pregnancy reaches 30% of women (3, 4). PPD is characterized by a depressed mood, loss of interest, sleep disturbances, psychomotor agitation or retardation, feelings of worthlessness, and even suicidal thoughts and behaviors in severe cases (5). Given the high prevalence and deleterious impact of PPD, the development of effective treatments is needed.

Treatment of PPD includes pharmacotherapy, psychotherapy, or both, which is consistent with the treatment recommended in guidelines for major depression (6). However, these treatments vary in efficacy (7–9), are cost (10), while adverse events are common (11, 12). Therefore, more effective and safer treatments for PPD are still needed. In this regard, acupuncture is perceived as an effective and safe alternative (13). A number of systematic reviews (SRs)/meta-analyses (MAs) have evaluated the efficacy of acupuncture for PPD, however their findings are inconsistent and the evidence credibility is unclear. Therefore, we provide a critical evaluation of SRs/MAs on the use of acupuncture to treat PPD.

## Methods

This study followed the methodology of the Cochrane Handbook and high-quality studies (14–16).

### Eligibility Criteria

The following eligibility criteria were used to screen studies: (a) SRs/MAs based on randomized controlled trials (RCTs) on the use of acupuncture to treat PPD; (b) participants diagnosed with PPD by a recognized guideline; (c) interventions included acupuncture therapy or acupuncture plus conventional medication (CM), while the control group was treated with CM, CM plus acupuncture, sham acupuncture, or other non-pharmacological therapy; (d) outcomes included the Hamilton Rating Scale for Depression (HAMD), Edinburgh Postnatal Depression Scale (EPDS), effective rate and estradiol levels. Repeated publications or studies lacking complete data were removed.

### Search Strategy

Embase, PubMed, Web of Science, Cochrane Library, CNKI, CBM, Wanfang, and VIP were searched for studies published between database creation and November 2021. The following search terms were applied: postpartum depression, acupuncture, meta-analysis, and systematic review. Table 1 presents the search strategy for the PubMed database.

**Table 1:** Search strategy for the PubMed database.

**Query**	**Search term**
# 1	Postpartum depression [Mesh]
# 2	Postpartum depression[Title/Abstract] OR postnatal depression[Title/Abstract] OR post-partum depression[Title/Abstract] OR post-natal depression[Title/Abstract] OR post natal depression[Title/Abstract]
# 3	#1 OR #2
# 4	Acupuncture[Mesh]
# 5	Acupuncture[Title/Abstract] OR pharmacoacupuncture[Title/Abstract] OR acupotomy[Title/Abstract] OR acupotomies[Title/Abstract] OR pharmacopuncture[Title/Abstract] OR needle[Title/Abstract] OR needling[Title/Abstract] OR dry-needling[Title/Abstract] OR body-acupuncture[Title/Abstract] OR electroacupuncture[Title/Abstract] OR electro-acupuncture[Title/Abstract] OR auricular acupuncture[Title/Abstract]
# 6	#4 OR #5
# 7	Meta-analysis as Topic[Mesh]
# 8	Systematic review[Title/Abstract] OR meta-Analysis[Title/Abstract] OR meta-analysis [Title/Abstract] OR meta-analyses[Title/Abstract] OR meta-analysis [Title/Abstract]
# 9	#7 OR #8
# 10	#3 AND #6 AND #9

### Data Collection and Extraction

Two independent evaluators screened abstracts and titles, and then assessed potentially eligible full texts for final inclusion. Disagreements were resolved through discussion with a third independent reviewer. The following data were extracted from included studies: first author, year of publication, country, sample size, interventions, outcomes, quality assessment methods, and summary estimates of effect.

### Quality Assessment

Two independent evaluators assessed the methodological quality of SR/MA using the Assessment of Methodological Quality of Systematic Evaluation 2 (AMSTAR-2) (17). AMSTAR-2 consists of 16 items, each with three possible answers, i.e., “yes,” “partially yes,” or “no.” When up to one non-critical item does not meet the requirements, the methodological quality is considered “high”; when more than one non-critical item does not meet the requirements, the methodological quality is considered “medium”; when one critical item does not meet the requirements, the methodological quality is considered “low” and when more than one critical item do not meet the requirements, the methodological quality is deemed “very low” (17).

Two independent evaluators used the Grade of Recommendation, Assessment, Development and Evaluation (GRADE) (18) to assess the quality of evidence for each outcome indicator. GRADE ranks the evidence according to risk of bias, indirectness, imprecision, inconsistency, and publication bias. Each outcome measure is rated on four levels, i.e., “high,” “moderate,” “low,” or “very low” (18).

### Data Synthesis and Presentation

A narrative synthesis was used in this overview. The characteristics and results of each SR/MA as well as results from AMSTAR 2 were summarized by tabulation. The GRADE evidence profile and summary of findings table were generated using the GRADE pro GDT online software.

## Results

### Study Selection

**Figure 1:**
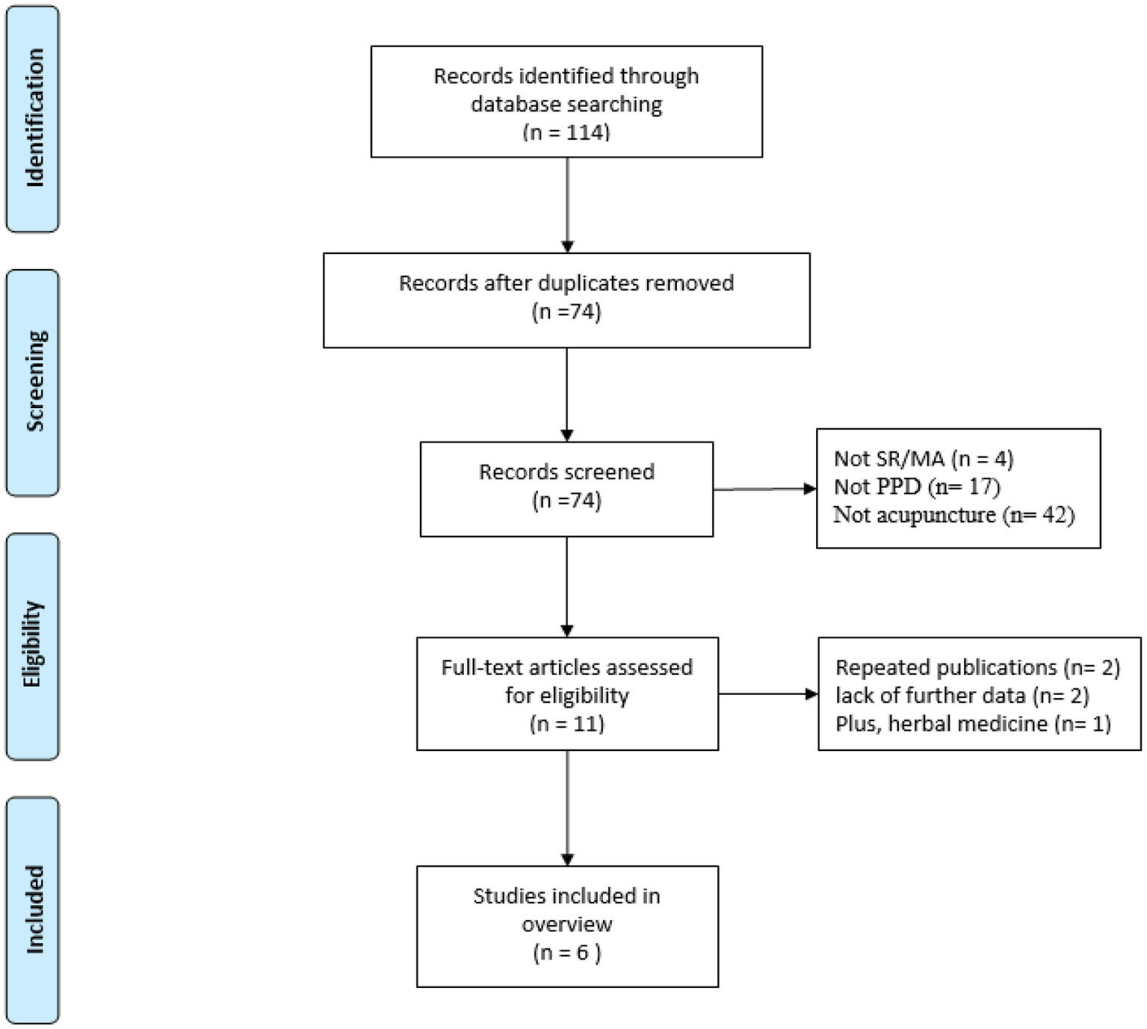
Flow diagram of the literature selection.

The literature search identified 114 articles, of which 40 duplicates were removed. Titles and abstracts of 74 articles were screened, and 63 articles were subsequently excluded. The full text of the remaining 11 articles was read and five articles were excluded. Therefore, six papers were included in our analyses (19–25). The selection process is shown in Figure 1.

### Study Characteristics

All included studies were conducted in China and published within the last 5 years. All studies were MAs with significant differences in sample size (5–14 studies, 27–934 participants). The interventions in the experimental group were acupuncture, or a combination of acupuncture and CM, while the control interventions were CM and/or sham acupuncture. Details on study characteristics are reported in Table 2.

**Table 2:** Characteristics of the included studies.

**References**	**Country**	**Sample size**	**Treatment intervention**	**Control intervention**	**Quality assessment**	**Conclusion**
Tong et al. (19)	China	12 (877)	AT; AT+CM	ST; CM	Cochrane criteria	Acupuncture has shown benefit in improving some symptoms of PPD, although the evidence is still inconclusive. High-quality studies are needed to confirm the effectiveness of acupuncture for PPD.
Li et al. (20)	China	8 (517)	AT	ST; CM	Cochrane criteria	Acupuncture treatment significantly improved HAMD scores, but had no significant effect on EPDS, clinical response, or serum estradiol levels.
Li et al. (21)	China	9 (653)	AT; AT+CM	ST; CM	Cochrane criteria	Acupuncture appears to be beneficial for PPD, however, the evidence is inconclusive. To confirm the effectiveness of acupuncture in PPD, further high-quality RCTs are needed.
Cao et al. (22)	China	13 (899)	AT	CM	Cochrane criteria	This study found no statistical difference between acupuncture and control groups in reducing HAMD scores and improving clinical effectiveness. Further studies are needed to validate these findings.
Wang et al. (23)	China	14 (934)	AT; AT+CM	ST; CM	Cochrane criteria	Acupuncture is effective in the treatment of PPD, but more high-quality and large sample size RCTs are needed to provide high-quality evidence.
Pang and Shi (24)	China	5 (279)	AT; AT+CM	ST; CM	Jadad	Acupuncture is as effective as CM and more effective than placebo to treat PPD. Acupuncture is safe and effective, although patients might experience fainting and pain during the procedure.

*AT, acupuncture therapy; ST, sham acupuncture; CM, conventional medication*.

### Methodological Appraisal

All studies were rated as very low quality according to the AMSTAR-2 criteria. The key factors contributing to lower methodological quality were item 2 (only one review registered a protocol), item 4 (half of the studies did not provide a search strategy), and item 7 (all reviews did not provide a list of excluded studies). Detailed assessment results of AMSTAR-2 are shown in Table 3.

**Table 3:** AMSTAR-2 assessment results.

**References**	**AMSTAR-2**																**Overall quality**
	**Q1**	**Q2**	**Q3**	**Q4**	**Q5**	**Q6**	**Q7**	**Q8**	**Q9**	**Q10**	**Q11**	**Q12**	**Q13**	**Q14**	**Q15**	**Q16**	
Tong et al. (19)	Y	PY	Y	Y	Y	Y	N	Y	Y	Y	Y	Y	Y	Y	Y	Y	CL
Li et al. (20)	Y	PY	Y	PY	Y	Y	N	Y	Y	Y	Y	Y	Y	Y	Y	Y	CL
Li et al. (21)	Y	Y	Y	Y	Y	Y	N	Y	Y	Y	Y	Y	Y	Y	Y	Y	CL
Cao et al. (22)	Y	PY	Y	Y	Y	Y	N	Y	Y	Y	Y	Y	Y	Y	Y	Y	CL
Wang et al. (23)	Y	PY	Y	PY	Y	Y	N	Y	Y	Y	Y	Y	Y	Y	Y	Y	CL
Pang and Shi (24)	Y	PY	Y	PY	Y	Y	N	Y	Y	N	Y	Y	Y	Y	Y	N	CL

*Y, Yes; PY, partial Yes; N, No; CL, Critically low; L, Low*.

### GRADE Evidence Quality Classification

A total of 19 outcome indicators were assessed. No indicator was deemed high, while two were moderate, 12 were low and five were very low quality of evidence. Risk of bias was the most common reason for downgrading the evidence, followed by inconsistency, imprecision, publication bias, and indirectness. Details are shown in Table 4.

**Table 4:** Certainty of evidences quality.

**References**	**Outcomes**	**Simple**	**Limitations**	**Inconsistency**	**Indirectness**	**Imprecision**	**Publication bias**	**Quality**
Tong et al. (19)	HAMD	MD −1.27 (−2.55, 0.01)	-1	-1	0	0	0	Low
	EPD	SMD −0.49 (−1.01, 0.02)	-1	-1	0	-1	-1	Very low
	Estradiol level	MD 63.99 (13.47, 114.51)	-1	-1	0	-1	-1	Very low
	Effect rate	RR 1.20 (1.09, 1.33)	-1	0	0	0	0	Moderate
Li et al. (20)	HAMD	SMD −1.08 (−2.11, −0.05)	-1	-1	0	0	0	Low
	Estradiol levels	SMD 1.96 (−0.01, 3.93)	-1	0	0	-1	-1	Very low
	Effect rate	RR 1.00 (0.89, 1.12)	-1	-1	0	0	0	Low
Li et al. (21)	HAMD	MD −1.38 (−3.40, 0.64)	-1	-1	0	0	0	Low
	EPDS	MD 1.08 (1.09, 3.26)	-1	-1	0	-1	-1	Very low
	Effective rate	RR 1.15 (1.06, 1.24)	-1	0	0	-1	0	Low
	Estradiol levels	MD 36.92 (23.14, 50.71)	-1	-1	0	0	0	Low
Cao et al. (22)	HAMD	MD 0.45 (−0.52,1.41)	-1	-1	0	0	0	Low
	EPDS	MD 0.55 (0.18, 0.92)	-1	0	0	-1	-1	Very low
	Effective rate	RR 0.93 (0.70, 1.23)	-1	-1	0	0	0	Low
	Estradiol levels	MD 0.20 (−0.19, 0.58)	-1	0	0	0	0	Moderate
Wang et al. (23)	HAMD	MD −1.27 (−2.55,0.01)	-1	-1	0	0	0	Low
	EPDS	MD −0.47 (−0.92, −0.03)	-1	0	0	-1	0	Low
	Estradiol levels	WMD 63.99 (13.39, 114.60)	-1	-1	0	0	0	Low
	Effective rate	OR 3.15 (2.19, 4.55)	-1	0	0	-1	0	Low
Pang and Shi (24)	HAMD	MD −1.03 (−2.58,0.52)	-1	-1	0	-1	-1	Very low
	Effective rate	RR 0.98 (0.84, 1.14)	-1	0	0	-1	-1	Very low

*RR, Risk Ratio; OR, odds ratio; SMD, SMD, standardized mean difference; WMD, Weighted Mean Difference; AT, acupuncture therapy; ST, sham acupuncture; CM, conventional medication; HAMD, Hamilton Rating Scale for Depression; EPDS, Edinburgh Postnatal Depression Scale. , The design of the experiment with a large bias in random, distributive hiding or blind; , The confidence interval overlaps less, the heterogeneity test P is very small, and the I2 is larger; , Confidence interval is not narrow enough; , Funnel graph asymmetry; , Fewer studies are included and there may be greater publication bias*.

### Description of Efficacy

All studies used the HAMD to assess the severity of depression, and one review (20) concluded that acupuncture treatment improved depressive symptoms more significantly than CM, however, five reviews (19, 21–24) showed no significant difference between the two groups. Four reviews (19, 21–23) reported the EPDS of acupuncture vs. CM, in which three reviews showed that acupuncture was more effective than the control group (21–23) and one review showed no significant difference (19). The effective rate was reported in all reviews. Three of which revealed that acupuncture was more effective than the control group (19, 21, 23) while the other three reviews found no difference (20, 22, 24). Estradiol levels were reported in five reviews (19–23), in which three reviews found a significant effect for acupuncture when compared to the control group (19, 21, 23) and one review found no difference (20, 22).

## Discussion

Acupuncture is routinely used in clinical therapy for PPD in China as a way to improve therapeutic effectiveness. Numerous SRs/MAs have evaluated the effectiveness of acupuncture for PPD, however, inconsistent results have been reported. In this context, a critical evaluation of different SRs/MAs and a summary of the scientific nature of the evidence is necessary (25). Furthermore, an overview can highlight deficiencies that need to be improved to guide future high-quality RCTs or SRs/MAs (26).

A total of six SR/MAs were included in this study, all of which were published in the past 5 years, suggesting that more researchers are beginning to study acupuncture as an alternative therapy for PPD. Nineteen outcome measures on the effectiveness of acupuncture to treat PPD were evaluated, and although most indicators suggested positive results, these were inconsistent. Furthermore, although most of the included studies suggested that acupuncture was effective as a treatment for PPD, most authors did draw firm conclusions due to the low methodological quality of evidence or the small size of included trials. Indeed, all reviews were considered to be of very low quality according to AMSTAR-2 criteria. Therefore, our analysis concluded that acupuncture might be an effective treatment for PPD, but such conclusion must be treated with caution due to limitations of the current evidence.

Over recent years, AMSTAR-2 has become the most widely used tool to evaluate the methodological quality of SRs/MAs. All included studies had more than one critical flaw, so that there is very low confidence in their results. The key factors contributing to this setting were item 2 (only one review registered a protocol), item 4 (half of the studies did not provide a search strategy), and item 7 (all reviews did not provide a list of excluded studies). It was found that study protocols contribute to increased transparency of the methodology used and improve the overall methodological quality of SRs/MAs (27). The absence of a specific search strategy can result in an unreproducible search process, which leads to significant bias in included and excluded studies, undermining the scientific validity of findings. Likewise, by not presenting a list of excluded studies, authors can concur to incorrect exclusion of key literature, undermining the rigor of the report. Therefore, future SRs/MAs should address these identified deficiencies to develop high-quality studies and thus provide high-quality evidence.

In this study, authors of the included SRs/MAs did not draw definitive conclusions. Indeed, after rating the evidence using the GRADE system, we found that the certainty of evidence was unsatisfactory, indicating that findings of the included SRs/MAs are uncertain. Although all SRs/MAs evaluated only RCTs, the certainty of evidence was limited owing to the risk of bias (lack of blinding and allocation concealment), inconsistency, imprecision, or publication bias. The results of the methodological quality evaluation of RCTs showed that there is room for addressing random, distributed hidden or blind biases. Nevertheless, we must acknowledge that there are specificities of acupuncture therapy (inability to blind physicians and patients) that make the implementation of RCTs challenging. Improved standardization and precision of acupuncture techniques and procedures are urgently needed, as only a rigorously designed and implemented RCT can reduce the risk of bias and therefore assess the effectiveness of interventions (28).

To our knowledge, this is the first overview of SRs/MAs summarizing the current evidence on the use of acupuncture to treat PPD. The methodological and evidence qualities of the included SRs/MAs may help to inform evidence-based decision-making and guide future high-quality studies. However, our study presents some limitations. First, the quality analysis demonstrated numerous methodological flaws in the performance of SRs/MAs, and the evidence quality was not satisfactory, making it impossible to draw firm conclusions about the use acupuncture for PPD. Second, the rapid growth in the number of SRs/MAs highlights challenges faced by healthcare decision-makers and researchers in keeping up with the evidence. This overview found that there were typically a large number of low-quality SRs/MAs. To help evidence-based practice, there is an urgent need for high-quality SRs/MAs that do not overlap and are up to date. Furthermore, widely used AMSTAR-2 tool and GRADE system are subjective evaluation tools, therefore the accuracy of assessments can vary. To mitigate this limitation, quality assessments were performed by two independent authors.

## Conclusion

Acupuncture might be beneficial for PPD. However, due to limitations of the current evidence and inconsistent findings, further studies are needed to provide strong evidence to draw definitive conclusions.

## Data Availability Statement

The original contributions presented in the study are included in the article/supplementary material, further inquiries can be directed to the corresponding authors.

## Author Contributions

XH and QF conceived the study and drafted the manuscript. LM, RJ, RG, and XZ help with the implementation of research. FQ and LZ provided guidance on the overview methodology. LM revised the manuscript. All authors read, critically reviewed, and approved the final manuscript as submitted.

## Funding

This work was funded by Jiangxi Provincial Department of Science and Technology Key Research and Development Program General Project (No. 20192BBGL70037) and Changshu Science and Technology Development Plan Project (No. CS202136).

## Conflict of Interest

The authors declare that the research was conducted in the absence of any commercial or financial relationships that could be construed as a potential conflict of interest.

## Publisher's Note

All claims expressed in this article are solely those of the authors and do not necessarily represent those of their affiliated organizations, or those of the publisher, the editors and the reviewers. Any product that may be evaluated in this article, or claim that may be made by its manufacturer, is not guaranteed or endorsed by the publisher.

## Abbreviations

PPD: postpartum depression
SR: Systematic review
MA: Meta-analysis
AMSTAR-2: Assessing the Methodological Quality of Systematic Reviews 2
GRADE, Grading of Recommendations, Assessment, Development: and Evaluation
RCTs: Randomized clinical trials
CM: conventional medication
HAMD: Hamilton Depression Scale
EPDS: Edinburgh Postnatal Depression Scale.
